# Stroke Avoidance for Children in REpública Dominicana (SACRED): Protocol for a Prospective Study of Stroke Risk and Hydroxyurea Treatment in Sickle Cell Anemia

**DOI:** 10.2196/resprot.7491

**Published:** 2017-06-02

**Authors:** Neelum D Jeste, Luisanna M Sánchez, Gabriela S Urcuyo, Melissa E Bergés, Judy P Luden, Susan E Stuber, Teresa S Latham, Rafael Mena, Rosa M Nieves, Russell E Ware

**Affiliations:** ^1^ Cincinnati Children's Hospital Medical Center Cancer and Blood Diseases Institute Cincinnati, OH United States; ^2^ Universidad Iberoamericana Santo Domingo Dominican Republic; ^3^ Centro de Obstetricia y Ginecología Santo Domingo Dominican Republic; ^4^ Hospital Infantil Robert Reid Cabral Santo Domingo Dominican Republic

**Keywords:** Dominican Republic, hydroxyurea, sickle cell anemia, stroke, transcranial Doppler

## Abstract

**Background:**

In the Dominican Republic, where the burden of sickle cell anemia (SCA) is high, many children lack access to routine screening and preventative care. Children with SCA are at risk for stroke, an event that leads to significant morbidity and mortality. In the United States, screening via transcranial Doppler (TCD) identifies children with SCA at highest stroke risk, allowing early intervention with blood transfusions. The need for indefinite transfusions for primary stroke prevention limits their practicality in limited-resource countries. Hydroxyurea has been shown to lower TCD velocities and to prevent conversion from conditional (170 to 199 cm/sec) to abnormal (greater than or equal to 200 cm/sec) velocities. In resource-limited settings, implementation of a TCD screening program, coupled with hydroxyurea therapy, could reduce the burden of SCA and stroke.

**Objective:**

The aims of the Stroke Avoidance for Children in REpública Dominicana (SACRED) trial are (1) to screen children with SCA for stroke risk using TCD and to determine the prevalence of elevated velocities in a cross-sectional sample; (2) to identify clinical and laboratory correlates of elevated velocities; and (3) to obtain longitudinal data on the natural history of TCD velocities and to measure therapeutic effects of hydroxyurea.

**Methods:**

This prospective trial, designed and conducted by Cincinnati Children’s Hospital Medical Center (CCHMC) and Hospital Infantil Robert Reid Cabral (HIRRC) with Centro de Obstetricia y Ginecología, includes a baseline cross-sectional epidemiological survey of the distribution of TCD velocities across a large cohort of children with SCA in the Dominican Republic. Children with conditional velocities are eligible to begin protocol-directed hydroxyurea if laboratory criteria are met. The treatment schedule begins with a fixed-dose of approximately 20 mg/kg/day for 6 months, after which it escalates to maximum tolerated dose (MTD). All participants undergo longitudinal annual TCD evaluation, while those on hydroxyurea have semi-annual evaluations during the 3-year study period. Data are collected using an Internet-based Research Electronic Data Capture (REDCap) system with forms translated into Spanish; both remote and on-site monitoring are used.

**Results:**

To date, 122 children with SCA have enrolled in SACRED including 85 (69.7%, 85/122) with normal, 29 (23.8%, 29/122) with conditional, 5 (4.1%, 5/122) with abnormal, and 3 (2.5%, 3/122) with inadequate TCD velocities. Of the 29 children with conditional TCD velocities, 17 (59%, 17/29) have initiated hydroxyurea per protocol, with plans for escalation to MTD.

**Conclusions:**

The SACRED trial will provide novel epidemiologic data about the prevalence of children with SCA and increased stroke risk in the Dominican Republic. The study also includes an investigation of the impact of hydroxyurea at MTD on elevated TCD velocities, as well as clinical and laboratory parameters. The design and implementation of SACRED reflect a successful international institutional partnership, one that features local capacity building and training in research methods and clinical care. The trial’s results have important implications for screening and prevention of primary stroke in children with SCA living in resource-limited settings.

**Trial Registration:**

ClinicalTrials.gov NCT02769845; https://www.clinicaltrials.gov/ct2/show/NCT02769845 (Archived by WebCite at http://www.webcitation.org/6qf6n0Egh)

## Introduction

Sickle cell anemia (SCA) is one of the most common inherited red blood cell disorders. Its prevalence is highest in sub-Saharan Africa, but there are also significant disease burdens in the Americas, India, Mediterranean region, and the Caribbean including Jamaica and the Dominican Republic [1]. SCA is associated with high morbidity and mortality, especially in limited-resource settings. Stroke is one of the most devastating clinical events to occur in children with SCA and can lead to considerable morbidity and early mortality. The frequency of primary stroke in children with homozygous hemoglobin S (HbSS), the most common and severe form of SCA, is approximately 5% to 10% [2,3].

Many pediatric sickle cell programs in the United States and Europe use transcranial Doppler (TCD) ultrasound screening to identify children at risk for developing primary stroke. TCD is a means of measuring blood velocity in the circle of Willis. The standard evaluation for stroke risk includes interrogation of the major intracranial vessels in both hemispheres including the middle cerebral artery, anterior cerebral artery, bifurcation of the middle cerebral and anterior cerebral arteries, distal internal carotid artery, posterior cerebral artery, and top of the basilar. In children with SCA, the time-averaged maximum velocity (TAMV) is recorded and normal velocities less than 170 cm/sec are associated with lowest stroke risk, while conditional velocities (170 to 199 cm/sec) and abnormal TCD velocities (200 cm/sec or greater) are associated with increased risk and highest risk, respectively [4]. Adams et al demonstrated that TCD could effectively be used to screen pediatric patients with SCA and found the relative risk of stroke was 44 times greater among patients with TCD velocities above 200 cm/sec [3,4]. The Stroke Prevention Trial in Sickle Cell Anemia (STOP) and subsequent STOP II trials demonstrated that children with abnormal velocities must receive chronic blood transfusions indefinitely to reduce the risk of primary stroke [5,6].

Several centers throughout the world have successfully utilized TCD screening, proving its feasibility in various patient populations [7-12]. TCD screening examinations are typically performed as early as 2 to 3 years of age in children with SCA and then annually thereafter. The natural history is variable, but some patients present with abnormal velocities while most young patients start in the normal or conditional range, and then over time may convert to the abnormal range. In a retrospective study, these increases were more likely in children less than 10 years of age, with 23% of conditional velocities converting to abnormal over an 18-month time period [13]. Young age, low oxygen saturation, severity of anemia, and low levels of fetal hemoglobin are among the demographic and clinical variables reported as correlates of elevated TCD velocities [9,14,15]. In contrast, inheritance of alpha-thalassemia trait has been described in several studies as a protective factor [16-18], whereas the impact of concomitant *glucose-6-phosphate dehydrogenase* (G6PD) deficiency on TCD velocities yields mixed findings [19,20]. In resource-limited settings where access to TCD is limited, identification of additional risk factors could be important for prioritizing patients who warrant early screening.

Hydroxyurea is a disease-modifying medication that induces fetal hemoglobin production [21], reduces the frequency of painful vaso-occlusive episodes [22], and lowers TCD velocities [23,24]. Two prospective multicenter phase 3 clinical trials funded by the National Institutes of Health (NIH) investigated the efficacy of hydroxyurea versus transfusions for stroke prevention in SCA; 90% secondary stroke prevention was observed (NCT00122980) [25] while 100% primary stroke prevention was achieved in children with abnormal TCD velocities (NCT01425307) [26]. A third international multicenter NIH-funded trial investigated the efficacy of hydroxyurea for children with conditional TCD velocities, and demonstrated a significant reduction in mean velocity of 15 cm/sec with no conversions to abnormal velocities and no primary stroke events [27].

The use of hydroxyurea for children with SCA is attractive in limited-resources settings because of its safety, ease of oral administration, and low cost, while its long-term risks appear to be relatively small [28]. Chronic blood transfusions are effective for stroke prevention in SCA but not without challenges including cost, alloimmunization, infection, iron overload, and limited supply in many parts of the world. Accordingly, implementation of TCD screening that is coupled to hydroxyurea treatment represents an important option for resource-limited areas. We previously designed a research protocol (EXTEND, NCT02556099) that utilizes hydroxyurea treatment for both primary and secondary stroke prevention in Jamaica, where chronic blood transfusions are not feasible [29]. The current Stroke Avoidance for Children in REpública Dominicana (SACRED) protocol (NCT02769845) focuses on broad TCD screening and hydroxyurea treatment for prevention of primary stroke, while highlighting another international collaboration within the Caribbean as a prototype for successful research partnerships throughout the world. The trial will provide novel epidemiologic and treatment data for children with SCA in the Dominican Republic, with significant implications for screening and stroke prevention in resource-limited settings. The purpose of this paper is to describe the trial’s implementation, design, and preliminary results.

## Methods

### Identifying the Knowledge Gap

In the Dominican Republic, an estimated 6% to 8% of the population has the sickle trait, while 0.12% of the population has homozygous SCA (HbSS). Since there is no formal newborn hemoglobinopathy screening program in the country, these numbers are estimates and based on anecdotal data. TCD screening is costly and primarily available to patients in the private sector. The prevalence of elevated TCD velocities in this population is unknown, as is the prevalence of clinical or genetic factors that might influence cerebral blood flow and stroke risk.

Hospital Infantil Robert Reid Cabral (HIRRC) is a large children’s hospital and referral center in Santo Domingo, the capital city of the Dominican Republic. Approximately 5.82% (64/1100) registered SCA patients have had overt stroke and receive chronic blood transfusions that are costly and difficult to maintain (unpublished data). The early identification of children with elevated TCD velocities, identification of variables that may predict stroke risk, and early intervention with hydroxyurea would therefore have significant public health implications. Hydroxyurea is available, but not utilized by most patients with SCA. The current clinical practice in the Dominican Republic is to offer hydroxyurea at around 15 mg/kg/day to families who can afford the daily medication, which costs US $1 per capsule. Preliminary data suggest safety and efficacy at a fixed dose in reducing vaso-occlusive crises in this population [30]. However, evaluation of escalation to maximum tolerated dose (MTD) is warranted when the goal is stroke prevention, since improved laboratory results are observed at higher treatment doses [31].

### Study Design and Objectives

**Figure 1:**
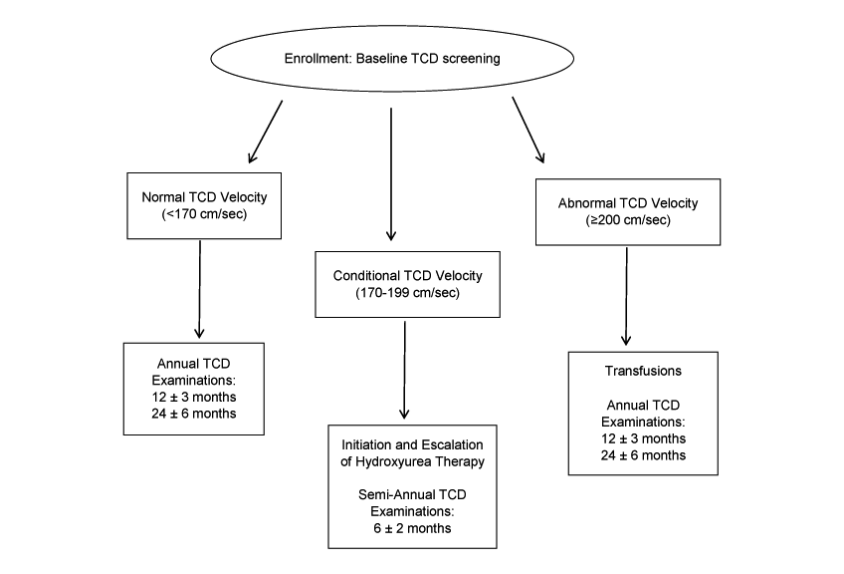
Study design.

SACRED involves a 3-part prospective study design which includes (1) baseline TCD evaluation; (2) longitudinal TCD evaluation; and (3) treatment if warranted (Figure 1).

#### Baseline Transcranial Doppler Evaluation

The baseline evaluation portion of SACRED involves obtaining TCD examinations on children with SCA between ages 3 to 15 years and followed at HIRRC. Up to 500 participants may be enrolled, but projected enrollment is 250 to 300 children over a 12-month period. All patients, including those who are already on hydroxyurea or monthly transfusion therapy (whether for stroke or other clinical indications) are included in study recruitment and TCD screening, to obtain an accurate 1-year cross-sectional description of velocities in the current SCA patient population. Additional baseline assessments will identify potential modifiers that influence cerebral blood flow including age, sex, medical history, hemoglobin concentration, and fetal hemoglobin, as well as genetic variants like G6PD deficiency, beta-globin haplotype, and alpha-thalassemia trait.

#### Longitudinal Transcranial Doppler Evaluation

For the longitudinal aspect of SACRED, participants undergo serial TCD examinations to help define the natural history of cerebrovascular disease in this population. Children on protocol-directed hydroxyurea therapy will undergo TCD every 6 months, while all other participants will have an annual examination.

#### Protocol-Directed Hydroxyurea Therapy

Children with conditional velocities between 170 to 199 cm/sec are eligible for protocol-directed hydroxyurea therapy, while those with abnormal velocities (200 cm/sec or greater) commence transfusion therapy per current practice guidelines at the clinical site. All participants who are already on chronic transfusion therapy at the time of enrollment, regardless of TCD result, may elect to remain on transfusion therapy per local clinical practice. SACRED participants will be followed until a common study termination date, defined as 3 years from the first participant’s initiation of hydroxyurea (Figure 1).

### Study Objectives and Hypotheses

The first objective of the SACRED trial is to screen a cohort of children with SCA in the Dominican Republic for stroke risk using TCD and to determine the prevalence of normal, conditional, and abnormal velocities in a cross-sectional sampling of children. Our hypothesis is the distribution of TCD velocities in a cross-sectional sampling of Dominican children will approximate that of prior studies in North America with a distribution of approximately 70% normal, 15% conditional, 10% abnormal, and 5% inadequate [32,33].

The second objective is to identify clinical and laboratory correlates of TCD velocities in a cohort of Dominican children such as age, prior hydroxyurea exposure, level of anemia, fetal hemoglobin, and genetic modifiers such as alpha-thalassemia trait and beta-globin haplotypes. We hypothesize that children with elevated TCD velocities will be younger, lack prior hydroxyurea exposure, have lower total hemoglobin and fetal hemoglobin levels, and higher levels of hemoglobin S (HbS) compared to participants with normal velocities. Beta-globin haplotypes will reveal a mixed genetic inheritance and alpha-thalassemia trait will be protective against elevated velocities.

Our third objective is to obtain longitudinal data on the natural history of TCD velocities in this patient cohort and to measure the effects of therapeutic intervention on TCD velocities, specifically hydroxyurea for conditional TCD velocities and transfusions for abnormal velocities. Given the potential neuroprotective effects of hydroxyurea, we anticipate a lower rate of conversion from conditional to abnormal than the 23% described in a previous observational study [13].

### Protocol Training

Training sessions for the local study team occurred remotely and on-site. Study personnel completed online Human Subjects Protection training via NIH training modules available in Spanish. An on-site investigators’ meeting occurred 6 months prior to study activation in which Cincinnati Children’s Hospital Medical Center (CCHMC) team members conducted protocol training, as well as training on informed consent, TCD, laboratory collection, hydroxyurea dosing calculator, pharmacy storage, adverse event reporting, and the Research Electronic Data Capture (REDCap) database. The Data Management Center (DMC) provided additional REDCap training in-person and remotely via Skype. Study coordinators utilized a training environment in the REDCap system in which they entered data for all case report forms on mock patients, which were verified and queried by the study monitor. Upon completion of REDCap training, coordinators were provided with an official certificate and access to the database. TCD training occurred over several months, with initial hands-on training by the CCHMC TCD coordinator. The local examiners then completed practice exams with upload and remote verification by the TCD coordinator. An official certificate was provided when the TCD coordinator verified that each local examiner had met certification criteria. SACRED Medical Coordinating Center (MCC) representatives from CCHMC were present for study activation and initial enrollment in July 2016.

### Study Setting

Participant recruitment, enrollment, TCD screening, and medical examinations occur at the primary clinical site at HIRRC. All evaluations are conducted by local team members who have completed the training as detailed above. Two study assessments—brain magnetic resonance imaging/magnetic resonance angiogram (MRI/MRA) and urine pregnancy tests for post-menarchal females on hydroxyurea therapy—occur at a partnering site, Centro de Obstetricia y Ginecología, also located in Santo Domingo. CCHMC serves as both the MCC and the DMC for the trial. SACRED was approved by the CCHMC institutional review board (IRB) on March 16, 2016, as well as 2 ethics boards in the Dominican Republic: Comité del Centro Nacional de Investigación en Salud Materno Infantil (CENISMI, through HIRRC) on April 29, 2016 and Consejo Nacional de Bioética en Salud (CONABIOS, National board) on June 28, 2016.

### Participant Recruitment and Enrollment

Children seen in the hematology clinic at HIRRC are invited to participate in SACRED. The informed consent document is signed by a parent or legal guardian, with assent as required by the local ethics board for all children 10 years and older. Consent includes acknowledgment of storage of genetic material. Inclusion criteria include (1) pediatric patients with severe forms of SCA (HbSS or HbS beta^0^-thalassemia); and (2) between 3 and 15 years of age at the time of enrollment. There are no exclusion criteria applicable to the baseline or longitudinal TCD screening portion of SACRED, but for participants with conditional TCD velocities, the following criteria disqualify them from treatment with hydroxyurea: (1) known medical condition making participation ill-advised (eg, acute or chronic infectious disease, known allergy to hydroxyurea, or malignancy); or (2) pregnancy. Participants with abnormal baseline laboratory values, defined as hemoglobin less than 6.0 gm/dL, absolute reticulocyte count (ARC) less than 100 x 10^9^/L with a hemoglobin less than 8.0 gm/dL, absolute neutrophil count (ANC) less than 1.0 x 10^9^/L, platelet count less than 80 x 10^9^/L, or elevated serum creatinine are temporarily excluded from starting hydroxyurea until improvement of the affected laboratory parameter into the acceptable range.

### Study Procedures and Treatment

For the schedule of evaluations, see Table 1. All enrolled participants undergo baseline TCD evaluation, medical history, physical examination, and laboratory analysis. The laboratory analysis includes complete blood count (CBC) with differential, reticulocyte count, serum chemistry, hemoglobin electrophoresis, and special studies to include saved serum and collection of blood onto Whatman Flinders Technology Associates cards (FTA) to be sent in batches to CCHMC for genetic analysis including testing for alpha-thalassemia trait, G6PD deficiency, and beta-globin haplotype. A child identified to have an abnormal velocity (200 cm/sec or greater) is scheduled for a repeat TCD within 3 weeks, and if confirmed to be abnormal, initiates erythrocyte transfusions per local standard of care. Children who are not treated with hydroxyurea per SACRED undergo annual evaluation (Figure 1).

**Table 1:** Schedule of study evaluations.

Evaluation	Enrollment	Year 1^a^	Year 2^b^
Medical history^c^	Yes	Yes	Yes
Prior/concomitant medications^c^	Yes	Yes	Yes
Physical examination^c^	Yes	Yes	Yes
Labs (complete blood count with differential, reticulocyte count)^c^	Yes	Yes	Yes
Hemoglobin electrophoresis	Yes	No	No
Hemoglobin F level^d^	Yes	Yes	Yes
TCD^e^ examination^f^	Yes	Yes	Yes
Special studies^g^	Yes	No	No
Brain MRI/MRA^h^ for participants on hydroxyurea only	Yes	No	Yes

^a^Year 1 is 12 months plus or minus 3 months.

^b^Year 2 is 24 months plus or minus 6 months.

^c^Participants on hydroxyurea will undergo these evaluations monthly until reaching maximum tolerated dose (MTD) and then quarterly after MTD is reached.

^d^Quarterly for participants on hydroxyurea.

^e^TCD: transcranial Doppler.

^f^Every 6 months for participants on hydroxyurea.

^g^Special studies includes specimens for genomic DNA analysis and serum biomarkers. Will be additionally collected at study exit for children on hydroxyurea.

^h^MRI/MRA: magnetic resonance imaging/magnetic resonance angiogram.

Children identified to have a conditional TCD (170 to 199 cm/sec) are eligible for protocol-directed hydroxyurea treatment. Additional evaluations for hydroxyurea-treated participants include collection of saliva (DNA Genotek Inc., Ottawa, Canada) to obtain DNA, urine pregnancy test (if applicable), and a brain MRI/MRA around the time of treatment initiation. Brain MRI/MRA images are additionally obtained at study exit for children on hydroxyurea to grade vasculopathy, using previously reported techniques [34] and to evaluate treatment effect. The non-contrast technique includes whole-brain imaging of sagittal T1, axial T1, coronal and axial fluid-attenuated inversion recover (FLAIR), T2-weighted, and diffusion-weighted images. Participants are evaluated clinically and sedation is provided if deemed necessary and parental consent obtained. The sequences are uploaded onto a secure research cloud that deidentifies images and are stored on a CCHMC research server for central review. Participants are aware that the MRI is for research purposes and real-time results are not provided.

Hydroxyurea is purchased locally and provided as generic 500 mg capsules. A hydroxyurea dosing calculator, previously described [35], is available on the SACRED website and provides recommended target dose based on a participant’s weight, current laboratory values, and previous dose. During the first 6 months of treatment, participants are administered hydroxyurea at a fixed dose of 20.0 plus or minus 5.0 mg/kg/day. The fixed dose of 20.0 mg/kg/day was selected to ensure hydroxyurea will be tolerated without excessive hematological toxicities; a decision made in conjunction with the local investigators. The large variation in starting dose is due to limitations with having only one available 500 mg capsule size locally. Average daily dosing, in which smaller children take medication on alternate days or skipping days of the week to reach targeted total weekly dose, does not permit precise dosing but has been used previously with success [31,36]. Capsules may be crushed and mixed into liquid for children with difficulty swallowing. During the fixed-dose phase, children undergo monthly study visits that include interval medical history, adverse event reporting, physical examination, and laboratory evaluation with CBC/differential and reticulocyte count. Every 3 months, fetal hemoglobin levels, serum chemistries, and urine pregnancy tests (if applicable) are obtained. After 6 months, hydroxyurea will be increased to MTD as defined by hematological toxicity, to achieve a target ANC of 2.0-4.0 x 10^9^/L. Hydroxyurea dose escalation will occur at 8-week intervals, in increments of 2.5 to 5.0 mg/kg/day. After reaching MTD, study visits occur every 3 months. During all phases of study treatment, medication is temporarily suspended should hematological toxicity, defined as ANC less than 1.0 x 10^9^/L, hemoglobin less than 7.5 gm/dL with an ARC less than 100 x 10^9^/L, ARC less than 80 x 10^9^/L with hemoglobin less than 8.5 gm/dL, or platelet count less than 80 x 10^9^/L occur. The dose escalation and toxicity criteria are listed in Table 2. Complications of hydroxyurea therapy and neurological events are collected at interval visits as part of adverse event reporting. Medication adherence is assessed at each visit by parental report and asking participants to bring the medication bottles so the local study team can count the number of returned capsules.

**Table 2:** Hydroxyurea dose escalation and toxicity criteria.

Toxicity	Parameter	Escalation criteria	Dose-limiting toxicity
Neutropenia	ANC^a^ (x10^9^/L)	> 4.0	<1.0 x 10^9^/L
Anemia	Hemoglobin (gm/dL)	> 6.5	Hb^b^ <7.5 gm/dL unless ARC^c^ >100 x 10^9^/L
Reticulocytopenia	ARC (x10^9^/L)	> 150	ARC <80 x 10^9^/L unless Hb >8.5 gm/dL
Thrombocytopenia	Platelets (x10^9^/L)	> 150	<80 x 10^9^/L

^a^ANC: absolute neutrophil count.

^b^Hb: hemoglobin.

^c^ARC: absolute reticulocyte count.

Study data are collected and managed using the REDCap electronic data system [33]. REDCap is a secure, Web-based application designed to support data capture for research studies, providing a platform for data entry and validation, audit trails for tracking data manipulation, and automated export procedures for data analysis. The system uses low bandwidth, rendering it suitable for research in low-income countries, as well as multilingual capabilities including Spanish. Data are entered into the electronic data record directly from the clinical site. Each enrolled participant is assigned a study identification (ID) number, which allows deidentified information to be collected. The DMC reviews data for accuracy and completeness via remote and on-site monitoring. Quality assurance monitoring is performed on the data at standard time intervals per the study’s Data Safety Monitoring Plan. Standard database reports, generated monthly, include enrollment, withdrawal, cumulative toxicities, and serious adverse events.

### Statistical Analyses

No sample size calculation was performed because the study utilizes a convenience sample. Descriptive analysis of the data from TCD screening will be performed. TCD velocities will be measured longitudinally and summary statistics such as mean, standard deviation, and median for the change of TCD measurements from baseline to study exit will be reported. The primary endpoints of SACRED are changes in TCD velocities over time as a measure of treatment response for participants on hydroxyurea and to evaluate natural history of TCD velocities for those not on treatment. Secondary endpoints will include hydroxyurea-related toxicities and clinical and laboratory correlates of TCD velocities. For patients on treatment, the highest TAMV for each time period along with the baseline values will be analyzed using repeated measures analysis of variance (MANOVA). Participants who are non-adherent to dosing or who are unable to continue hydroxyurea treatment secondary to treatment-associated toxicities will be analyzed according to intention-to-treat. However, hydroxyurea discontinuation dates and adherence information will be recorded for secondary analysis.

Baseline labs will be compared to exit studies for parameters such as hemoglobin, fetal hemoglobin, ANC, ARC, and platelets using descriptive statistics and comparative *t* tests. For analyses of genetic modifiers, single nucleotide polymorphisms (SNPs) from either candidate genes or whole exome sequencing methods will be tested for their association with the phenotypes of interest.

## Results

**Figure 2:**
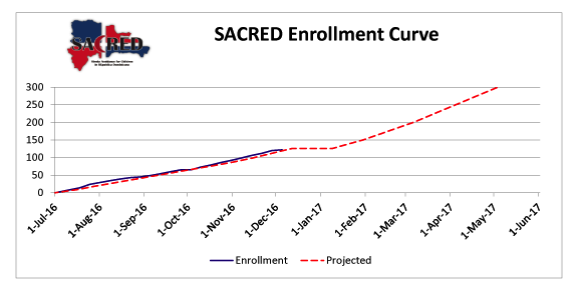
Enrollment curve.

Enrollment began on July 18, 2016. At present, a total of 122 participants have been enrolled (Figure 2). All participants who were approached and met the inclusion criteria have consented for SACRED. The categorical results, shown in Table 3, include 85 (69.7%, 85/122) participants with a normal TCD, while 29 (23.8%, 29/122) have a conditional, 5 (4.1%, 5/122) have an abnormal, and 3 (2.5%, 3/122) have an inadequate TCD. Of the 29 children with conditional velocities, 17 (59%, 17/29) have already initiated protocol-directed hydroxyurea therapy. Of the participants, 22 (18.0%, 22/122) children were already on hydroxyurea at time of enrollment, 2 (9%, 2/22) of whom had conditional velocities and were eligible for treatment on study-directed dosing. The initial baseline screening phase will continue for approximately 12 months from study activation. The common study termination date is 3 years after the first participant began hydroxyurea, which is scheduled for August 2019.

**Table 3:** Participant status (N=122).

Characteristic		Participants, n
Total enrolled		122
Participants with completed TCD^a^ exam		122
TCD exam classification		
	Abnormal	5
	Conditional	29
	Normal	85
	Inadequate	3
	TCD not yet reviewed	0
Hydroxyurea treatment status		
	On hydroxyurea at study entry	22
	Eligible for protocol-directed hydroxyurea	20
	Ineligible for hydroxyurea	0
	Temporarily ineligible for hydroxyurea	1
	Initiated hydroxyurea	17
	Declined hydroxyurea/alternate therapy^b^	8
	Withdrawn after treatment initiation^c^	0
	Currently on hydroxyurea	17
Completed study		0

^a^TCD: transcranial Doppler.

^b^Transfusion due to history of stroke or abnormal TCD.

^c^Includes deceased.

## Discussion

### Principal Findings

SACRED is the first prospective trial investigating stroke risk among children with SCA in the Dominican Republic, a country where the burden of disease is high. During the first 5 months of the trial, we have observed an excellent rate of patient accrual, which we anticipate will continue, given the reported interest among the local patient population and strong visit adherence within the hydroxyurea treatment group. Our initial data suggest a slightly higher prevalence of conditional, but lower prevalence of abnormal TCD results than anticipated, which could reflect differences in baseline status, previous treatment, or potentially genetics. Data from the United States demonstrate the distribution of TCD velocities to be approximately 70% normal, 15% conditional, 10% abnormal, and 5% inadequate [32,33].

The SACRED trial is also the first study to evaluate the effects of hydroxyurea on decreasing stroke risk in children with SCA living in the Dominican Republic. The potential for using hydroxyurea in this setting is valuable, as it is currently being used for specific clinical indications including vaso-occlusive crises, acute chest syndrome, or repeated priapism. Adding primary stroke prophylaxis to the indications for hydroxyurea could have important implications for reducing the incidence of this devastating clinical event and for preventing its subsequent morbidity and clinical burden. If proven to be beneficial, the goal would be for hydroxyurea to be available nationally at an affordable cost for all at-risk children with SCA. The importance of prospective data regarding its use and safety in the Dominican population are thus crucial to ensure proper accessibility and utilization of this medication.

In addition to the many challenges inherent to conducting an international clinical research trial related to the protocol and data collection, there is a more subtle concern about changing the local standard of care by imposing US-based methods. For example, current clinical practice at the local site is to dose hydroxyurea at 15 mg/kg/day, while SACRED dosing aims to start at approximately 20 mg/kg/day with escalation to MTD. We have explained that our goal is to study the safety and efficacy of higher doses based on published data [37], without suggesting that the site’s prior clinical approach was incorrect. Another challenge involves creating dosing regimens with a single 500 mg capsule size, and thus far we have observed exceptional patient compliance even with this alternate dosing schedule.

### Conclusion

SACRED is a prospective trial that will yield valuable public health information pertaining to stroke screening and risk among Dominican children with SCA. Further, SACRED will establish local capacity to conduct high-quality research through training and experience with both TCD screening and hydroxyurea therapy among local clinicians. The knowledge and experience gained from SACRED will advance research expertise at the local site and improve clinical care for children with SCA in this country, with the opportunity to expand to other Caribbean nations.

## Abbreviations

ANC: absolute neutrophil count
ARC: absolute reticulocyte count
CBC: complete blood count
CCHMC: Cincinnati Children’s Hospital Medical Center
DMC: Data Management Center
HbS: hemoglobin S
HIRRC: Hospital Infantil Robert Reid Cabral
G6PD: glucose-6-phosphate dehydrogenase
MCC: Medical Coordinating Center
MRA: magnetic resonance angiogram
MRI: magnetic resonance imaging
MTD: maximum tolerated dose
NIH: National Institutes of Health
REDCap: Research Electronic Data Capture
SACRED: Stroke Avoidance for Children in REpública Dominicana
SCA: sickle cell anemia
STOP: Stroke Prevention Trial in Sickle Cell Anemia
TAMV: time-averaged maximum velocity
TCD: transcranial Doppler

## References

[1] Piel FB, Patil AP, Howes RE, Nyangiri OA, Gething PW, Dewi M, Temperley WH, Williams TN, Weatherall DJ, Hay SI (2013). Global epidemiology of sickle haemoglobin in neonates: a contemporary geostatistical model-based map and population estimates. Lancet.

[2] Balkaran B, Char G, Morris JS, Thomas PW, Serjeant BE, Serjeant GR (1992). Stroke in a cohort of patients with homozygous sickle cell disease. J Pediatr.

[3] Ohene-Frempong K, Weiner SJ, Sleeper LA, Miller ST, Embury S, Moohr JW, Wethers DL, Pegelow CH, Gill FM (1998). Cerebrovascular accidents in sickle cell disease: rates and risk factors. Blood.

[4] Adams R, McKie V, Nichols F, Carl E, Zhang DL, McKie K, Figueroa R, Litaker M, Thompson W, Hess D (1992). The use of transcranial ultrasonography to predict stroke in sickle cell disease. N Engl J Med.

[5] Adams RJ, McKie VC, Hsu L, Files B, Vichinsky E, Pegelow C, Abboud M, Gallagher D, Kutlar A, Nichols FT, Bonds DR, Brambilla D (1998). Prevention of a first stroke by transfusions in children with sickle cell anemia and abnormal results on transcranial Doppler ultrasonography. N Engl J Med.

[6] Adams RJ, Brambilla D, Optimizing Primary Stroke Prevention in Sickle Cell Anemia (STOP 2) Trial Investigators (2005). Discontinuing prophylactic transfusions used to prevent stroke in sickle cell disease. N Engl J Med.

[7] Makani J, Kirkham FJ, Komba A, Ajala-Agbo T, Otieno G, Fegan G, Williams TN, Marsh K, Newton CR (2009). Risk factors for high cerebral blood flow velocity and death in Kenyan children with Sickle Cell Anaemia: role of haemoglobin oxygen saturation and febrile illness. Br J Haematol.

[8] McCarville MB, Goodin GS, Fortner G, Li C, Smeltzer MP, Adams R, Wang W (2008). Evaluation of a comprehensive transcranial doppler screening program for children with sickle cell anemia. Pediatr Blood Cancer.

[9] Bernaudin F, Verlhac S, Arnaud C, Kamdem A, Chevret S, Hau I, Coïc L, Leveillé E, Lemarchand E, Lesprit E, Abadie I, Medejel N, Madhi F, Lemerle S, Biscardi S, Bardakdjian J, Galactéros F, Torres M, Kuentz M, Ferry C, Socié G, Reinert P, Delacourt C (2011). Impact of early transcranial Doppler screening and intensive therapy on cerebral vasculopathy outcome in a newborn sickle cell anemia cohort. Blood.

[10] Enninful-Eghan H, Moore RH, Ichord R, Smith-Whitley K, Kwiatkowski JL (2010). Transcranial Doppler ultrasonography and prophylactic transfusion program is effective in preventing overt stroke in children with sickle cell disease. J Pediatr.

[11] Colombatti R, Meneghetti G, Ermani M, Pierobon M, Sainati L (2009). Primary stroke prevention for sickle cell disease in north-east Italy: the role of ethnic issues in establishing a transcranial Doppler screening program. Ital J Pediatr.

[12] Melo HA, Barreto-Filho JA, Prado RC, Cipolotti R (2008). Transcranial doppler in sickle cell anaemia: evaluation of brain blood flow parameters in children of Aracaju, Northeast-Brazil. Arq Neuropsiquiatr.

[13] Hankins JS, Fortner GL, McCarville MB, Smeltzer MP, Wang WC, Li C, Ware RE (2008). The natural history of conditional transcranial Doppler flow velocities in children with sickle cell anaemia. Br J Haematol.

[14] Hokazono M, Silva G, Silv EM, Braga JA (2011). Results from transcranial Doppler examination on children and adolescents with sickle cell disease and correlation between the time-averaged maximum mean velocity and hematological characteristics: a cross-sectional analytical study. Sao Paulo Med J.

[15] Lagunju I, Sodeinde O, Brown B, Akinbami F, Adedokun B (2014). Transcranial Doppler ultrasonography in children with sickle cell anemia: clinical and laboratory correlates for elevated blood flow velocities. J Clin Ultrasound.

[16] Adams RJ, Kutlar A, McKie V, Carl E, Nichols FT, Liu JC, McKie K, Clary A (1994). Alpha thalassemia and stroke risk in sickle cell anemia. Am J Hematol.

[17] Hsu LL, Miller ST, Wright E, Kutlar A, McKie V, Wang W, Pegelow CH, Driscoll C, Hurlet A, Woods G, Elsas L, Embury S, Adams RJ, Stroke Prevention Trial (STOP) and the Cooperative Study of Sickle Cell Disease (CSSCD) (2003). Alpha thalassemia is associated with decreased risk of abnormal transcranial Doppler ultrasonography in children with sickle cell anemia. J Pediatr Hematol Oncol.

[18] Figueiredo M, Kerbauy J, Gonçalves MS, Arruda V, Saad S, Sonati M, Stoming T, Costa FF (1996). Effect of alpha-thalassemia and beta-globin gene cluster haplotypes on the hematological and clinical features of sickle-cell anemia in Brazil. Am J Hematol.

[19] Bernaudin F, Verlhac S, Chevret S, Torres M, Coic L, Arnaud C, Kamdem A, Hau I, Grazia NM, Delacourt C (2008). G6PD deficiency, absence of alpha-thalassemia, and hemolytic rate at baseline are significant independent risk factors for abnormally high cerebral velocities in patients with sickle cell anemia. Blood.

[20] Flanagan JM, Frohlich DM, Howard TA, Schultz WH, Driscoll C, Nagasubramanian R, Mortier NA, Kimble AC, Aygun B, Adams RJ, Helms RW, Ware RE (2011). Genetic predictors for stroke in children with sickle cell anemia. Blood.

[21] Platt OS, Orkin SH, Dover G, Beardsley GP, Miller B, Nathan DG (1984). Hydroxyurea enhances fetal hemoglobin production in sickle cell anemia. J Clin Invest.

[22] Charache S, Terrin ML, Moore RD, Dover GJ, Barton FB, Eckert SV, McMahon RP, Bonds DR (1995). Effect of hydroxyurea on the frequency of painful crises in sickle cell anemia. Investigators of the multicenter study of hydroxyurea in sickle cell anemia. N Engl J Med.

[23] Zimmerman SA, Schultz WH, Burgett S, Mortier NA, Ware RE (2007). Hydroxyurea therapy lowers transcranial Doppler flow velocities in children with sickle cell anemia. Blood.

[24] Kratovil T, Bulas D, Driscoll MC, Speller-Brown B, McCarter R, Minniti CP (2006). Hydroxyurea therapy lowers TCD velocities in children with sickle cell disease. Pediatr Blood Cancer.

[25] Ware RE, Helms RW, SWiTCH Investigators (2012). Stroke with transfusions changing to hydroxyurea (SWiTCH). Blood.

[26] Ware RE, Davis BR, Schultz WH, Brown RC, Aygun B, Sarnaik S, Odame I, Fuh B, George A, Owen W, Luchtman-Jones L, Rogers ZR, Hilliard L, Gauger C, Piccone C, Lee MT, Kwiatkowski JL, Jackson S, Miller ST, Roberts C, Heeney MM, Kalfa TA, Nelson S, Imran H, Nottage K, Alvarez O, Rhodes M, Thompson AA, Rothman JA, Helton KJ, Roberts D, Coleman J, Bonner MJ, Kutlar A, Patel N, Wood J, Piller L, Wei P, Luden J, Mortier NA, Stuber SE, Luban NL, Cohen AR, Pressel S, Adams RJ (2016). Hydroxycarbamide versus chronic transfusion for maintenance of transcranial doppler flow velocities in children with sickle cell anaemia-TCD With Transfusions Changing to Hydroxyurea (TWiTCH): a multicentre, open-label, phase 3, non-inferiority trial. Lancet.

[27] Hankins JS, McCarville MB, Rankine-Mullings A, Reid ME, Lobo CL, Moura PG, Ali S, Soares DP, Aldred K, Jay DW, Aygun B, Bennett J, Kang G, Goldsmith JC, Smeltzer MP, Boyett JM, Ware RE (2015). Prevention of conversion to abnormal transcranial Doppler with hydroxyurea in sickle cell anemia: a phase III international randomized clinical trial. Am J Hematol.

[28] Brawley OW, Cornelius LJ, Edwards LR, Gamble VN, Green BL, Inturrisi C, James AH, Laraque D, Mendez M, Montoya CJ, Pollock BH, Robinson L, Scholnik AP, Schori M (2008). National Institutes of Health Consensus Development Conference statement: hydroxyurea treatment for sickle cell disease. Ann Intern Med.

[29] Rankine-Mullings AE, Little CR, Reid ME, Soares DP, Taylor-Bryan C, Knight-Madden JM, Stuber SE, Badaloo AV, Aldred K, Wisdom-Phipps ME, Latham T, Ware RE (2016). EXpanding Treatment for Existing Neurological Disease (EXTEND): an open-label phase II clinical trial of hydroxyurea treatment in sickle cell anemia. JMIR Res Protoc.

[30] Svarch E, Machín S, Nieves RM, Mancia de Reyes AG, Navarrete M, Rodríguez H (2006). Hydroxyurea treatment in children with sickle cell anemia in Central America and the Caribbean countries. Pediatr Blood Cancer.

[31] Zimmerman SA, Schultz WH, Davis JS, Pickens CV, Mortier NA, Howard TA, Ware RE (2004). Sustained long-term hematologic efficacy of hydroxyurea at maximum tolerated dose in children with sickle cell disease. Blood.

[32] Adams RJ, Brambilla DJ, Granger S, Gallagher D, Vichinsky E, Abboud MR, Pegelow CH, Woods G, Rohde EM, Nichols FT, Jones A, Luden JP, Bowman L, Hagner S, Morales KH, Roach ES (2004). Stroke and conversion to high risk in children screened with transcranial Doppler ultrasound during the STOP study. Blood.

[33] Adams RJ, McKie VC, Carl EM, Nichols FT, Perry R, Brock K, McKie K, Figueroa R, Litaker M, Weiner S, Brambilla D (1997). Long-term stroke risk in children with sickle cell disease screened with transcranial Doppler. Ann Neurol.

[34] Helton KJ, Adams RJ, Kesler KL, Lockhart A, Aygun B, Driscoll C, Heeney MM, Jackson SM, Krishnamurti L, Miller ST, Sarnaik SA, Schultz WH, Ware RE, SWiTCH Investigators (2014). Magnetic resonance imaging/angiography and transcranial Doppler velocities in sickle cell anemia: results from the SWiTCH trial. Blood.

[35] McGann PT, Tshilolo L, Santos B, Tomlinson GA, Stuber S, Latham T, Aygun B, Obaro SK, Olupot-Olupot P, Williams TN, Odame I, Ware RE (2016). Hydroxyurea therapy for children with sickle cell anemia in Sub-Saharan Africa: rationale and design of the REACH trial. Pediatr Blood Cancer.

[36] Kinney TR, Helms RW, O'Branski EE, Ohene-Frempong K, Wang W, Daeschner C, Vichinsky E, Redding-Lallinger R, Gee B, Platt OS, Ware RE (1999). Safety of hydroxyurea in children with sickle cell anemia: results of the HUG-KIDS study, a phase I/II trial. Pediatric Hydroxyurea Group. Blood.

[37] Ware RE, Aygun B (2009). Advances in the use of hydroxyurea. Hematology Am Soc Hematol Educ Program.

